# Discriminative capacity of the Spanish version of the Inventory of Depression and Anxiety Symptoms-II (IDAS-II) for detecting DMS-5 specific disorders and poor quality of life in a clinical sample

**DOI:** 10.1186/s12955-024-02270-x

**Published:** 2024-07-18

**Authors:** Manuel Sanchez-Garcia, Carmen Díaz-Batanero, Ana De la Rosa-Cáceres

**Affiliations:** 1 Department of Clinical and Experimental Psychology, Facultad de Ciencias de la Educación, University of Huelva, Universidad de Huelva, Huelva, 21071 Spain; 2 Research Center for Natural Resources, Health and the Environment, University of Huelva, Universidad de Huelva, Huelva, 21071 Spain

**Keywords:** Inventory of Depression and Anxiety Symptoms II, Cutoffs, Quality of life, Diagnoses, Emotional disorders, Internalizing.

## Abstract

**Background:**

Emotional problems can be evaluated using categorical approaches to guide treatment choices focused on targeting specific disorders, or dimensional approaches to reduce symptom severity. Moreover, recent evidence points out the need to intervene in patients’ quality of life (QoL), which often remains low even after the remission of emotional problems. Thus, assessment instruments are needed to provide information on diagnosis, symptom severity, and QoL. The present study aimed to provide diagnostic and QoL cutoffs for the Inventory of Depression and Anxiety Symptoms-II (IDAS-II).

**Methods:**

273 patients recruited from mental health services in Huelva (Spain) completed the IDAS-II, Mini International Neuropsychiatric Interview, and Short Form-36 Health Survey. Receiver operating characteristic curve analyses were used to establish cutoff values. Diagnostic, balanced, and screening cutoffs were provided for each IDAS-II scale to detect corresponding diagnoses and poor QoL.

**Results:**

The specific IDAS-II scales Suicidality, Panic, Social Anxiety, Claustrophobia, and Traumatic Intrusions showed adequate discrimination values for their corresponding diagnoses (suicidal behavior disorder, panic disorder, social anxiety disorder, agoraphobia, and post-traumatic stress disorder, respectively). Both the General Depression and Dysphoria scales showed adequate ability to detect major depressive disorder. The IDAS-II scales showed a higher discrimination ability for Mental Health-related QoL, than for General Health-related QoL.

**Conclusions:**

The diagnostic and QoL cutoffs expand the clinical utility of the IDAS-II in clinical practice and research, making it a comprehensive, detailed, and versatile self-report tool. The IDAS-II allows for the assessment of emotional problems consistent with the dimensional, categorical, transdiagnostic, and QoL approaches.

**Supplementary Information:**

The online version contains supplementary material available at 10.1186/s12955-024-02270-x.

Depression and anxiety are the most prevalent mental disorders worldwide [1], with prevalences of 33.7% and 31.9%, respectively, during the COVID-19 pandemic [2]. These disorders are associated with more years lived with disability [1, 3], poor quality of life (QoL) [4, 5], and high rates of comorbidity with other internalizing disorders [6, 7] (e.g., obsessive-compulsive disorder [OCD], post-traumatic stress disorder [PTSD], and social anxiety disorder [SAD]).

Mood and anxiety disorders are commonly diagnosed using the categorical systems of the DSM [8] and ICD [9], which rely on the presence (versus absence) of certain symptoms. These systems unify diagnostic language, monitor prevalence statistics, and guide treatment choices, where each diagnosis has traditionally been treated with syndrome-specific treatments [10]. For example, Division 12 of the APA [11] provides a list of psychological treatments recommended for specific disorders, such as Behavioral Activation for depression. The DSM and ICD remain the most widely used diagnostic systems worldwide [12, 13].

Complementary dimensional approaches have emerged in recent decades, including Research Domain Criteria [14], Hierarchical Taxonomy of Psychopathology (HiTOP) [15], network theory [16], and Process-Based Therapy [17, 18]. Dimensional approaches conceptualize psychopathological constructs as a continuum of severity, especially focusing on symptoms, and guide treatment planning [10]. For instance, HiTOP describes symptom severity to aid in treatment selection [19, 20]. Additionally, clinicians can intervene in broader (spectra) or narrower (symptoms) levels of HiTOP depending on the patient’s needs. Interventions on spectra allow the treatment of comorbidities by focusing on common aspects of different disorders through transdiagnostic interventions [21]. Alternatively, clinicians can treat specific, clinically relevant symptoms [19]. In parallel, Process-Based Therapy tracks symptom changes [22] and network theory helps visualize the relationship between symptoms [16], guiding the development of personalized interventions [10, 23, 24].

Although dimensional approaches mainly focus on reducing symptom severity, recent evidence highlights the importance of improving patient QoL for several reasons [25–27]. First, depression and anxiety affect QoL [4, 25] more than do other chronic diseases [28]. Certain symptoms such as sad mood and lack of concentration are especially disabling, indicating the relevance of assessing the specific symptomatology of each patient [29, 30]. Second, QoL plays a crucial role in establishing a diagnosis, as a person must experience clinically significant distress or functional impairment due to their symptoms [8]. Finally, QoL predicts prolonged remission of depression more effectively than decreases in symptomatology [26, 27], suggesting that good QoL may serve as a protective factor against depressive episodes. Consequently, QoL has been incorporated as an outcome measure [31, 32], and some authors advocate integrating QoL measures into instruments assessing depression.

Given the importance of both categorical, and dimensional models and the growing concern about QoL, measurement instruments should be able to discriminate between disorders, assess symptom severity, and provide information about QoL. Furthermore, in transdiagnostic interventions, the symptoms of different disorders should be evaluated using the same timeframe and response format to avoid variance bias related to the method [33, 34]. One of the instruments that best addresses these requirements is the Inventory of Depression and Anxiety Symptoms-II (IDAS-II) [35]. The IDAS-II is widely used to assess depression [36], and has been recommended as an efficient transdiagnostic measure to assess internalizing symptoms [37]. This instrument has shown adequate psychometric properties in English, Turkish, Spanish, German, Romanian, and Swedish [35, 38–42]. Additionally, the IDAS-II has norms to interpret symptom severity [43, 44] and clinical cutoffs to differentiate between functional and dysfunctional samples, and between moderate and severe impairment [45]. However, diagnostic cutoffs to differentiate among clinical diagnoses are only available for the first version of the instrument (IDAS) [46], and cutoffs to discriminate poor QoL have not yet been provided.

The present study aims to expand the clinical utility of the IDAS-II by examining the accuracy of its scales in discriminating between the presence and absence of internalizing disorders such as major depressive disorder (MDD), generalized anxiety disorder (GAD), suicidal behavior disorder (SBD), panic disorder (PD), SAD, agoraphobia, and PTSD. Further, the explanatory capacity of the IDAS-II to determine poor QoL will be analyzed. Screening, diagnostic, and balance cutoffs to discriminate between the presence and absence of their corresponding mental disorders (e.g., the cutoff of the Panic IDAS-II scale to discriminate the presence of PD and between poor and average/good QoL will be determined. According to previous research, we expect to find: i) adequate values of discrimination (AUC ≥ .70 [47]) for the specific IDAS-II scales and their corresponding diagnosis; ii) broader IDAS-II scales of Dysphoria and General Depression will show greater AUC for MDD and GAD; and iii) General Depression and Dysphoria scores will show better explanatory ability to determine poor QoL [29, 30].

## Methods

### Participants and procedure

A total of 273 patients were selected from public and private mental health services in Huelva (Spain), between June 2020 and September 2023. Owing to the lack of a recent census of people undergoing treatment at mental healthcare centers, a non-probability systematic sampling procedure was applied. Patients consecutively cited for treatment during the study period were invited to participate, as long as they met the inclusion and exclusion criteria. Inclusion criteria were age 18–80 years and being under treatment at a mental health service during data collection. Exclusion criteria included having a medical or psychological disorder that disqualified them from taking the tests, such as acute psychotic disorders and severe or profound intellectual disabilities, or not signing the informed consent form.

The mean age was 39.96 years (*SD* = 15.43), and 74% were women. Of the participants, 2.6% had not completed primary education, 9.2% had completed primary education, 21.6% had completed secondary education, 45.5% had completed post-compulsory education, and 21.3% had completed university education. Regarding employment status, 45.5% of the respondents were employed. According to the DSM-5, 78.8% of the participants met the diagnostic criteria for at least one mental disorder, and 59.3% had diagnostic comorbidity. Table 1 shows the prevalence of diagnoses in the sample (Bipolar I Disorder [BID] and Bipolar II Disorder [BIID] were excluded because of their low prevalence rates [0.70% and 0.40%, respectively]). The most frequent diagnoses were GAD (50.18%) and MDD (46.52%). The highest comorbidity rates were found between GAD and MDD (29.67%), and between GAD and OCD (21.98%).

**Table 1:** Prevalence and comorbidities of the sample (*N* = 273)

	MDD	GAD	SBD	PD	SAD	Agoraphobia	PTSD	OCD
	*n* (%)	*n* (%)	*n* (%)	*n* (%)	*n* (%)	*n* (%)	*n* (%)	*n* (%)
Total prevalence	127 (46.52)	137 (50.18)	11 (4.06)	53 (19.41)	40 (14.71)	48 (17.58)	57 (20.88)	87 (31.87)
Women	100 (79.4)	110 (80.3)	8 (72.7)	44 (83.0)	28 (70.0)	40 (83.3)	47 (82.5)	62 (71.3)
Men	27 (21.3)	27 (19.7)	3 (27.3)	9 (17.0)	12 (30.0)	6 (12.5)	10 (17.5)	25 (28.7)
Mean Age (SD)	44.02 (14.76)	41.54 (14.66)	44.64 (11.42)	39.87 (14.43)	37.88 (12.35)	43.88 (13.60)	40.11 (13.77)	39.35 (13.47)
Comorbidities								
MDD	-	81 (59.12)	5 (45.45)	26 (49.05)	22 (55.00)	26 (54.16)	32 (56.14)	45 (51.72)
GAD	81 (63.77)	-	6 (54.54)	38 (71.69)	27 (67.50)	35 (72.91)	42 (73.18)	60 (68.96)
SBD	5 (3.93)	6 (4.37)	-	4 (7.54)	3 (8.5)	6 (12.50)	5 (8.77)	8 (9.19)
PD	26 (20.47)	38 (27.73)	4 (36.36)	-	14 (35.00)	23 (47.91)	17 (29.82)	27 (31.03)
SAD	22 (17.32)	27 (19.70)	3 (27.27)	14 (26.41)	-	26 (54.16)	19 (33.33)	21 (24.13)
Agoraphobia	26 (20.47)	35 (25.54)	6 (54.54)	23 (43.39)	26 (65.00)	-	22 (38.59)	24 (27.58)
PTSD	32 (25.19)	42 (30.65)	5 (45.45)	17 (32.07)	19 (47.50)	22 (45.83)	-	29 (33.33)
OCD	45 (35.43)	60 (43.79)	8 (72.72)	27 (50.94)	21 (52.50)	24 (50.00)	29 (50.87)	-

*Note* MDD = Major Depressive Disorder; GAD = Generalized Anxiety Disorder; SBD = Suicidal Behavior Disorder; PD = Panic Disorder; SAD = Social Anxiety Disorder; PTSD = Post-Traumatic Stress Disorder; OCD = Obsessive-Compulsive Disorder

A trained psychologist individually administered the instruments in the centers where the participants were recruited using paper-based methods. All participants were informed of their anonymous and voluntary participation in the study and provided written informed consent. An anonymized code was established for each patient to ensure that no identifying information was recorded anywhere. The custody of the interviews and the anonymized database complied with applicable data protection regulations. Participants received a voucher redeemable for gifts. This study and data management process was approved by the Bioethics Committee of the Province of Huelva (Junta de Andalucía, Spain) (No. 0275-N-21).

## Measures

### Inventory of Depression and Anxiety Symptoms-II

(IDAS-II; Spanish version; [39]) was used to assess internalizing symptoms. The IDAS-II is composed of 99 items rated on a 5-point scale (from 1 “*not at all*” to 5 “*extremely*”). Higher scores indicate higher symptom severity. The items are organized into 18 scales, in addition to General Depression (a scale composed of items from other scales). All scales assess specific symptoms of internalizing disorders except for Dysphoria and General Depression, which have a broad scope. General Depression provides an overall score for depression, whereas Dysphoria assesses the core symptoms of depression and anxiety disorders. The reliability values for the sample used in this study are listed in Table 2. Cronbach’s alpha coefficient ranged between .71 and .92, except for Lassitude scale (α = .69). Reliability values estimated using McDonald’s omega coefficient ranged between .70 and .92, except for Lassitude scale (ω = .68).

**Table 2:** Descriptive statistics and reliability of the measured scales (*n* = 273)

	Descriptive statistics			Reliability	
Scale	Minimum	Maximum	Mean (SD)	Cronbach’s alpha (α)	McDonald’s omega (ω)
**IDAS-II**					
General Depression	20	100	61.49 (15.19)	.87	.91
Dysphoria	10	50	32.56 (8.42)	.88	.88
Lassitude	6	30	15.89 (4.78)	.69	.68
Insomnia	6	30	18.15 (7.10)	.91	.91
Suicidality	6	30	9.86 (4.83)	.83	.86
Appetite Loss	3	15	6.71 (3.52)	.92	.92
Appetite Gain	3	15	6.49 (3.23)	.73	.76
Well Being (recode)	8	40	28.64 (7.06)	.89	.89
Ill Temper	5	25	13.09 (5.29)	.85	.85
Mania	5	25	14.06 (4.58)	.73	.74
Euphoria	5	25	8.63 (3.66)	.71	.70
Panic	8	40	20.79 (7.96)	.90	.90
Social Anxiety	6	30	15.18 (6.42)	.84	.85
Claustrophobia	5	25	10.57 (5.79)	.89	.89
Trauamtic Intrusions	4	20	11.04 (4.68)	.83	.83
Traumatic Avoidance	4	20	10.84 (3.88)	.73	.74
Checking	3	15	7.78 (3.26)	.78	.79
Ordering	5	25	11.46 (4.46)	.75	.73
Cleaning	7	35	12.67 (6.11)	.85	.85
**SF-36**					
Mental Health	0	100	32.33 (13.10)	.83	.85
General Health	0	100	37.23 (16.78)	.70	.77

### Mini-International Neuropsychiatric Interview

(MINI; Spanish version for DSM-5; [48–50]) was used to assess the diagnosis of mental disorders according to the DSM-5 criteria. Interviewer asks about a set of presence of symptoms using yes/no questions. According to the number of symptoms detected in each disorder, the scoring rules of the instrument specify which diagnoses are assigned to each patient. In the present study, the modules for MDD, SBD, BID, BIID, PD, agoraphobia, SAD, OCD, PTSD, and GAD were administered.

### Short Form-36 Health Survey

(SF-36; Spanish version; [51]) was used to assess QoL. The SF-36 consists of 36 items with multi-item scales that measure health status domains (Physical Functioning, Role-Physical, Bodily Pain, General Health, Vitality, Social Functioning, Role-Emotional, and Mental Health). Higher scores indicate a better health status. The present study used scores from mental and general health domains. The General Health score was calculated by summing recoded items 1, 11a, 11b, 11c, and 11d, and the Mental Health score was computed by summing recoded items 9b, 9c, 9d, 9f, and 9 h [52]. Mental Health assesses general mental health, including depression, anxiety, behavioral and emotional control, and overall positive affect. General Health assesses current health, future health prospects, and resistance to illness. In the present study, Cronbach’s alpha coefficient were .83 (Mental Health) and .70 (General Health), and McDonald’s omega coefficient were .85 (Mental Health) and .77 (General Health) (see Table 2).

## Data analysis

Missing values were found in 1.1% of respondents in the MINI and 0.37% of respondents in the SF-36. Cases with missing values were excluded from analyses using the pairwise procedure.

First, we provided descriptive statistics for the IDAS-II scales and the Mental Health and General Health domains of the SF-36. Internal consistency was assessed by estimating Cronbach’s alpha (α) and McDonald’s omega (ω).

Second, we applied receiver operating characteristic (ROC) curve analysis to examine the discrimination of the IDAS-II scales between the presence and absence of each internalizing DSM-5 diagnosis showing a prevalence higher than 4% in the sample, ensuring a minimum sample size of 10 patients for each category. ROC analysis evaluates the performance of the diagnostic classification (presence vs. absence of a disorder) by determining the sensitivity and specificity associated with each possible cutoff point [53]. Sensitivity corresponds to the proportion of people with the disorder correctly classified as having it and specificity corresponds to the proportion of people without the disorder correctly classified as not having it. Next, ROC analysis was applied to examine the discrimination of the IDAS-II scales between poor and average/good QoL. We applied a cutoff point of T-score ≤ 35 to the Mental Health and General Health QoL domain scores to differentiate between poor and average/good QoL. This criterion, indicating levels of the measure in the lower 7% of the population, is widely considered an indicator of clinical significance across commonly used measures [54, 55] in clinical practice and within research contexts to identify clinically significant impairments [56, 57]. To analyze the accuracy of the measure for classifying subjects according to reference standards (DSM-5 diagnosis and QoL level), we estimated the AUC [58], which reflects the ability of the test to discriminate between individuals with and without the diagnosis [59]. AUC values > .70 indicate poor discrimination, .70–.80 acceptable discrimination, .80–.90 excellent discrimination, and > .90 outstanding discrimination [47].

Finally, we estimated the cutoffs for the IDAS-II scales to discriminate their corresponding diagnoses (e.g., cutoff for the Panic scale in detecting PD). Considering the broad scope of the General Depression and Dysphoria, we developed cutoffs for these scales to predict both GAD and MDD. Cutoffs for the IDAS-II to discriminate between poor or average/good Mental Health and General Health-related QoL were also estimated. The cutoff values were estimated only when the AUC values were ≥ .70. Based on a previous study developed for the IDAS [46], we determined three cutoffs for each scale: *diagnostic* cutoff (more conservative) corresponding to the lowest score with a minimum specificity of .90; *screening* cutoff (more liberal) corresponding to the highest score with a minimum sensitivity of .90; and *balanced* cutoff (most useful in research contexts) optimal for discriminating between those who met the diagnostic criteria for a disorder and those who did not, corresponding to the score with the lowest difference between sensitivity and specificity. For each cutoff score, we computed sensitivity, specificity, and Youden’s index [60] (ranging between 0 and 1), with higher values indicating better discriminative capacity of the cutoffs. Finally, for cutoffs to discriminate diagnoses, we estimated the positive predictive value (PPV) (the probability of actually having a diagnosis when the test result is positive), negative predictive value (NPV) (the probability of not actually having the diagnosis when the test result is negative), LR+ (the ratio between the probability of a sick person testing positive and the probability of a healthy person testing positive, indicating how many times a sick person is more likely to test positive than a healthy person to test positive), and LR- (the ratio between the probability of an individual with a disease having a negative test and the probability of an individual without a disease having a negative test). That is, LR- indicates the proportion of healthy subjects who obtained a negative result) [61]. A 95% confidence interval was calculated for each value [62]. The confidence intervals of the likelihood ratios (LR) were calculated using the ‘Log method’ as described on page 109 of the text of Altman et al. [63]. The values for sensitivity, specificity, Youden’s index, PPV, NPV, LR+, and LR- of the MINI diagnoses to predict quality of life were obtained to compare them with the values obtained for the IDAS-II scales. The results of this analysis are presented as supplementary material. Analyses were conducted using SPSS 29.

## Results

The AUC values for each IDAS-II scale for predicting the DSM-5 diagnoses are shown in Table 3. Regarding the general IDAS-II scales, both General Depression and Dysphoria showed adequate discrimination for MDD (AUC = .78 and .76, respectively), whereas only General Depression showed adequate discrimination for GAD (AUC = .70). Adequate values of discrimination were also found for General Depression and Dysphoria in explaining SBD (AUC = .77 and.74, respectively) and SAD (AUC = .72 and .73), for Dysphoria in explaining PD (AUC = .70), and for General Depression in explaining PTSD (AUC = .70) and OCD (AUC = .70). Concerning the specific IDAS-II scales, we found adequate AUC values for Suicidality in predicting SBD (AUC = .84), Panic in predicting PD (AUC = .71), Social Anxiety in predicting SAD (AUC = .83), Claustrophobia in predicting Agoraphobia (AUC = .77), and Traumatic Intrusions in predicting PTSD (AUC = .76). In contrast, AUC values < .70 were found for Traumatic Avoidance in predicting PTSD (AUC = .63), and Checking (AUC = .64), Ordering (AUC = .62), and Cleaning (AUC = .56) in predicting OCD.

**Table 3:** AUC values [lower/upper 95% CI] for DSM-5 MINI diagnoses and SF-36 domains

	DSM-5 MINI Diagnoses								SF-36	
Scales	MDD	GAD	SBD	PD	SAD	Agoraphobia	PTSD	OCD	Mental H.	General H.
IDAS-II										
General D.	**.78 [.73/.84]**	**.70 [.64/.76]**	**.77 [.63/.91]**	.69 [.61/.77]	**.72 [.63/.81]**	.66 [.57/.75]	**.70 [.62/.77]**	**.70 [.63/.76]**	**.90 [.86/.94]**	**.74 [.68/.80]**
Dysphoria	**.76 [.70/.82]**	.69 [.63/.75]	**.74 [.60/.87]**	**.70 [.55/.71]**	**.73 [.64/.82]**	.66 [.57/.75]	.68 [.60/.75]	.69 [.63/.76]	**.87 [.83/.91]**	**.71 [.65/.78]**
Lassitude	.64 [.57/.70]	.65 [.58/.71]	.57 [.41/.72]	.63 [.54/.71]	.62 [0.58/.72]	.61 [.53/.69]	.62 [.55/.70]	.63 [.57/.70]	**.75 [.68/.82]**	**.72 [.66/.78]**
Insomnia	**.74 [.68/.80]**	.60 [.54/.67]	.62 [.44/.80]	.63 [.54/.71]	.63 [.54/.72]	.60 [.50/.69]	.64 [.55/.73]	.61 [.54/.69]	**.76 [.69/.82]**	.65 [.58/.71]
Suicidality	.68 [.62/.74]	.65 [.58/.71]	**.84 [.75/.93]**	.61 [.53/.70]	.69 [.59/.78]	.67 [.58/.75]	.68 [.60/.76]	.69 [.62/.76]	**.76 [.70/.82]**	**.71 [.64/.77]**
Ap. Loss	.66 [.59/.72]	.58 [.51/.65]	**.81 [.72/.91]**	.59 [.51/.68]	.57 [.47/.66]	.56 [.47/.65]	.65 [.57/.74]	.59 [.52/.67]	**.72 [.66/.79]**	.63 [.56/.70]
Ap. Gain	.51 [.44/.58]	.49 [.43/.56]	.36 [.24/.48]	.52 [.44/.61]	.60 [.51/.70]	.53 [.43/.63]	.56 [.47/.64]	.58 [.51/.66]	.59 [.52/.67]	.53 [.46/.60]
Well-B. (r.)	.67 [.60/.73]	**.70 [.64/.76]**	.67 [.47/.86]	.69 [.61/.77]	**.72 [.63/.81]**	.64 [.55/.73]	.62 [.55/.70]	.61 [.54/.68]	**.80 [.74/.85]**	.68 [.62/.74]
Ill Temper	.63 [.56/.69]	.63 [.56/.69]	.55 [.37/.72]	.62 [.54/.70]	.57 [.48/.66]	.60 [.52/.69]	.61 [.52/.69]	.67 [.60/.74]	**.75 [.68/.81]**	.61 [.54/.68]
Mania	.57 [.51/.64]	.60 [.54/.67]	.52 [.33/.71]	.65 [.57/.73]	.61 [.52/.71]	.58 [.49/.66]	.59 [.51/.67]	.67 [.60/.74]	.63 [.55/.71]	.61 [.54/.68]
Euphoria	.43 [.36/.50]	.45 [.38/.52]	.40 [.26/.55]	.49 [.41/.58]	.35 [.25/.44]	.44 [.36/.52]	.52 [.43/.60]	.55 [.48/.62]	.37 [.30/.45]	.44 [.37/.51]
Panic	**.73 [.67/.79]**	.69 [.63/.76]	.60 [.43/.77]	**.71 [.63/.78]**	.64 [.54/.73]	.67 [.58/.75]	.68 [.62/.75]	**.71 [.65/.78]**	**.81 [.75/.87]**	**.72 [.66/.78]**
Social Anx.	.61 [.54/.68]	.64 [.57/.71]	**.76 [.62/.89]**	.69 [.61/.76]	**.83 [.77/.89]**	**.72 [.64/.80]**	**.76 [.70/.82]**	.69 [.63/.76]	**.71 [.64/.77]**	.67 [.61/.73]
Claustroph.	.62 [.55/.69]	.57 [.51/.64]	.66 [.49/.84]	.62 [.54/.71]	.65 [.56/.74]	**.77 [.70/.85]**	.69 [.61/.77]	.58 [.51/.66]	**.70 [.63/.77]**	.67 [.61/.74]
Trauma. Int.	.65 [.59/.71]	.63 [.56/.69]	**.80 [.66/.93]**	.63 [.55/.71]	**.71 [.62/.80]**	.68 [.60/.76]	**.76 [.69/.83]**	**.72 [.65/.78]**	**.74 [.68/.81]**	**.72 [.66/.79]**
Trauma. Av.	.48 [.41/.55]	.47 [.41/.54]	**.74 [.61/.87]**	.54 [.46/.62]	.58 [.49/.68]	.60 [.51/.69]	.63 [.55/.72]	.56 [.49/.63]	.53 [.45/.60]	.48 [.40/.55]
Checking	.55 [.48/.62]	.61 [.54/.68]	.64 [.48/.79]	.61 [.53/.70]	.59 [.50/.68]	.60 [.51/.69]	.59 [.52/.67]	.64 [.57/.71]	.57 [.50/.65]	.59 [.52/.66]
Ordering	.52 [.45/.59]	.54 [.47/.61]	.43 [.27/.58]	.56 [.48/.65]	.55 [.46/.64]	.57 [.49/.66]	.60 [.52/.68]	.62 [.55/.69]	.50 [.42/.57]	.53 [.46/.60]
Cleaning	.57 [.51/.64]	.48 [.41/.55]	.61 [.41/.82]	.57 [.49/.66]	.55 [.45/.66]	.60 [.51/.69]	.59 [.50/.68]	.56 [.49/.68]	.55 [.48/.62]	.62 [.55/.68]

*Note * MDD = Major Depressive Disorder; GAD = Generalized Anxiety Disorder; SBD = Suicidal Behavior Disorder; PD = Panic Disorder; SAD = Social Anxiety Disorder; PTSD = Post-Traumatic Stress Disorder; OCD = Obsessive-Compulsive Disorder; General H. = General Health; Mental H. = Mental Health; General D. = General Depression; Ap. Loss = Appetite Loss; Ap. Gain = Appetite Gain; Well-B. (r.) = Well-Being (recode); Social Anx. = Social Anxiety; Claustroph. = Claustrophobia; Trauma. Int. = Traumatic Intrusions; Trauma. Av. = Traumatic Avoidance; AUC values ≥ 0.70 are shown in bold. Cells for convergent associations between IDAS-II scales and MINI DSM-5 diagnoses are shaded

The AUC values for each IDAS-II scale in predicting Mental Health and General Health are shown in Table 3. Six IDAS-II scales showed adequate discrimination for both Mental Health and General Health (with higher AUC values for Mental Health): General Depression (AUC = .74 and .90, respectively), Dysphoria (AUC = .71 and .87), Lassitude (AUC = .72 and .75), Suicidality (AUC = .71 and .76), Panic (AUC = .72 and .81), and Traumatic Intrusions (AUC = .72 and .74). Other six IDAS-II scales showed adequate AUC values only for predicting Mental Health: Insomnia (AUC = .76), Appetite Loss (AUC = .72), Well-Being (recoded) (AUC = .80), Ill Temper (AUC = .75), Social Anxiety (AUC = .71) and Claustrophobia (AUC = .70).

Table 4 shows the cutoff scores for the IDAS-II scales for predicting their corresponding diagnoses. For instance, the most adequate cutoff for the General Depression scale was for predicting MDD (AUC = .78). The balanced cutoff (≥ 66; percentile 58) showed adequate values of sensitivity (.693 [.61/.77]) and specificity (.767 [.70/.84]), and a high prediction of true diagnosis when the result was positive (PPV = .721; LR + = 2.97) and negative (NPV = .742; LR- = 0.40). The screening cutoff (≥ 53; percentile 34) showed high sensitivity (.913 [.86/.96]) and remarkable values of LR- (0.17) or NPV (only 13% of people with a score below the cutoff could be diagnosed with MDD), and an acceptable PPV (up to 61% of people diagnosed with MDD could truly have the disorder) and small LR+ (1.83). Regarding the diagnostic cutoff (≥ 77; percentile 85), 74% of people with a score equal to or greater than the cutoff will probably meet the criteria for MDD diagnosis. This diagnostic cutoff had a very high specificity (.911 [.87/.96]) and an acceptable NPV (.594). Concerning the specific scales, the higher AUC values corresponded to Suicidality for predicting SBD (AUC = .84) and Social Anxiety for predicting SAD (AUC = .83).

**Table 4:** Cutoffs of the IDAS-II scales and the utility of predicting DSM-5 MINI diagnoses

IDAS-II scale	Predicted DSM-5 diagnosis	AUC [95% CI]	Cutoff		Sensitivity	Specificity	J	PPV	NPV	LR+	LR-
General Depression	MDD	.78 [.73/.84]	diagnostic	76.5	.283 [.205/.361]	.911 [.865/.957]	.194 [.103/.285]	.735 [.611/.858]	.594 [.529/.658]	3.18[1.77/5.73]	0.79[0.70/0.89]
			balanced	65.5	.693 [.613/.773]	.767 [.698/.836]	.460 [.354/.566]	.721 [.642/.801]	.742 [.672/.812]	2.97[2.17/4.08]	0.40[0.30/0.53]
			screening	52.5	.913 [.864/.962]	.500 [.419/.581]	.413 [.318/.508]	.614 [.544/.683]	.869 [.796/.941]	1.83[1.54/2.17]	0.17[0.10/0.31]
	GAD	.70 [.64/.76]	diagnostic	75.5	.299 [.222/.376]	.904 [.854/.954]	.203 [.112/.294]	.759 [.644/.872]	.562 [.496/.627]	3.12[1.75/5.54]	0.78[0.69/0.88]
			balanced	61.5	.672 [.593/.751]	.662 [.582/.742]	.334 [.222/.446]	.667 [.588/.746]	.667 [.588/.747]	1.99[1.53/2.59]	0.50[0.38/0.65]
			screening	43.5	.920 [.875/.965]	.199 [.132/.266]	.119 [.038/.200]	.536 [.473/.600]	.711 [.568/.856]	1.15[1.04/1.27]	0.40[0.21/0.78]
Dysphoria	MDD	.76 [.70/.82]	diagnostic	40.5	.291 [.212/.370]	.918 [.873/.963]	.209 [.118/.300]	.755 [.635/.876]	.598 [0.534/.662]	3.55[1.93/6.51]	0.77[0.68/0.87]
			balanced	33.5	.709 [.630/.788]	.719 [.646/.792]	.428 [.320/.536]	.687 [.608/.766]	.739 [.667/.812]	2.52[1.90/3.35]	0.41[0.30/0.54]
			screening	26.5	.906 [.855/.957]	.425 [.345/.505]	.331 [.236/.426]	.578 [.510/.647]	.838 [.755/.922]	1.58[1.36/1.83]	0.22[0.13/0.39]
	GAD	.69 [.63/.75]									
			AUC value was insufficient for computing cutoff scores								
Suicidality	SBD	.84 [.75/.93]	diagnostic	15.5	.545 [.251/.839]	.900 [.864/.936]	.445 [.148/.742]	.188 [.052/.323]	.979 [.961/.997]	5.45[2.84/10.46]	0.51[0.26/0.97]
			balanced	11.5	.727 [.464/.990]	.723 [.669/.777]	.450 [.181/.719]	.100 [.034/.166]	.984 [.967/.999]	2.63[1.74/3.96]	0.38[0.14/0.99]
			screening	8.5	1 [.980/.999]	.569 [.509/.629]	.569 [.505/.631]	.089 [.039/.140]	1 [.999/1]	2.32[2.01/2.67]	0.00[0.00/**]
Panic	PD	.71 [.63/.78]	diagnostic	31.5	.208 [.099/.317]	.927 [.893/.961]	.135 [.020/.250]	.407 [.222/.592]	.829 [.782/.876]	2.85[1.41/5.77]	0.85[0.74/0.99]
			balanced	21.5	.755 [.639/.871]	.609 [.545/.673]	.364 [.231/.497]	.317 [.236/.399]	.912 [.866/.958]	1.93[1.54/2.42]	0.40[0.25/0.65]
			screening	12.5	.925 [.854/.996]	.218 [.163/.273]	.143 [.054/.232]	.222 [.167/.277]	.923 [.851/.996]	1.18 [1.07/1.31]	0.34[0.13/0.92]
Social Anxiety	SAD	.83 [.77/.89]	diagnostic	23.5	.450 [.296/.604]	.927 [.894/.960]	.377 [.219/.535]	.514 [.350/.681]	.908 [.870/.944]	6.16[3.48/10.93]	0.59[0.45/0.79]
			balanced	16.5	.875 [.773/.977]	.690 [.630/.750]	.565 [.446/.684]	.327 [.238/.416]	.970 [.944/.996]	2.82[2.25/3.53]	0.18[0.08/0.41]
			screening	13.5	.900 [.807/.993]	.530 [.466/.594]	.430 [.317/.543]	.247 [.178/.319]	.969 [.938/.999]	1.92[1.61/2.27]	0.19[0.07/0.48]
Claustro-phobia	Agora-phobia	.77 [.70/.85]	diagnostic	18.5	.375 [.238/.512]	.920 [.885/.955]	.295 [.154/.436]	.500 [.337/.663]	.873 [.831/.916]	4.69[2.64/8.32]	0.68[0.54/0.85]
			balanced	11.5	.708 [.579/.837]	.720 [.661/.779]	.428 [.287/.569]	.351 [.255/.445]	.920 [.880/.960]	2.53[1.92/3.34]	0.41[0.26/0.64]
			screening	5.5	.938 [.870/1]	.333 [.271/.395]	.271 [.179/.363]	.231 [.172/.290]	.962 [.919/1]	1.41[1.25/1.58]	0.19[0.06/0.57]
Traumatic Intrusions	PTSD	.76 [.69/.83]	diagnostic	16.5	.351 [.227/.475]	.903 [.864/.942]	.254 [.124/.384]	.488 [.335/.642]	.841 [.793/.888]	3.62[2.11/6.20]	0.72[0.59/0.87]
			balanced	11.5	.772 [.663/.881]	.634 [.570/.698]	.406 [.280/.532]	.358 [.273/.442]	.913 [.868/.958]	2.11[1.68/2.64]	0.36[0.22/0.59]
			screening	8.5	.912 [.838/.986]	.449 [.383/.515]	.361 [.262/.460]	.304 [.235/.373]	.951 [.909/.993]	1.66[1.43/1.91]	0.20[0.08/0.46]

*Note * MDD = Major Depressive Disorder; GAD = Generalized Anxiety Disorder; SBD = Suicidal Behavior Disorder; PD = Panic Disorder; SAD = Social Anxiety Disorder; PTSD = Post-Traumatic Stress Disorder; J = Youden’s index; PPV = positive predictive value; NPV = negative predictive value; LR + = positive likelihood ratio; LR- = negative likelihood ratio. *** = 227507.762

The cutoff scores for the IDAS-II scales for predicting poor Mental Health and General Health-related QoL are presented in Table 5. General Depression had the highest discrimination value for Mental Health (AUC = .90). The balanced cutoff (≥ 54; percentile 35) showed high values of sensitivity (.833 [.78/.88]) and specificity (.855 [.77/.94]). The diagnostic cutoff (≥ 61; percentile 48) corresponded to a very high specificity (.942 [.89/1]) and adequate sensitivity (.695 [.63/.76]). In turn, the screening cutoff (≥ 50; percentile 23) showed a higher sensitivity (.916 [.88/.95]). Sensitivity and specificity values computed for each of the IDAS-II scores predicting poor Mental Health (T-score ≤ 35) are higher than the same values computed for equivalent MINI diagnosis (see supplementary Table S1).

**Table 5:** Cutoffs of the IDAS-II scales and utility of predicting SF-36 domains (General Health and Mental Health)

IDAS-II scale	Predicted SF-36 domain	AUC [95% CI]	Cutoff		Sensitivity	Specificity		J	PPV	NPV	LR+		LR-
General Depression	Mental Health	.90 [.86/.94]	diagnostic	60.5	.695[.632/.758]	.942[.887/.997]	.637[.553/.721]		.972[.946/.999]	.512[.425/.599]	11.98[4.61/31.15]	0.32[0.26/0.40]	
			balanced	53.5	.833[.782/.884]	.855[.772/.938]	.688[.590/.786]		.944[.911/.978]	.635[.537/.733]	5.75[3.23/10.22]	0.20[0.14/0.27]	
			screening	49.5	.916[.878/.954]	.638[.525/.751]	.554[.434/.674]		.882[.838/.925]	.721[.608/.833]	2.53[1.85/3.47]	0.13[0.08/0.21]	
Dysphoria		.87 [.83/.91]	diagnostic	32.5	.665[.600/.730]	.913[.846/.980]	.578[.485/.671]		.957[.924/.991]	.481[.395/.566]	7.64[3.54/16.52]	0.37[0.30/0.45]	
			balanced	29.5	.793[.737/.849]	.768[.668/.868]	.561[.447/.675]		.910[.867/.952]	.558[.458/.658]	3.42[2.21/5.28]	0.27[0.20/0.36]	
			screening	24.5	.906[.866/.946]	.522[.404/.640]	.428[.303/.553]		.848[.800/.896]	.654[.528/.779]	1.90[1.48/2.44]	0.18[0.11/0.29]	
Lassitude		.75 [.68/.82]	diagnostic	18.5	.374[.307/.441]	.913[.846/.980]	.287[.193/.381]		.927[.870/.983]	.331[.265/.398]	4.30[1.96/9.42]	0.69[0.60/0.78]	
			balanced	14.5	.675[.611/.739]	.710[.603/.817]	.385[.260/.510]		.873[.820/.925]	.426[.336/.517]	2.33[1.59/3.41]	0.46[0.36/0.59]	
			screening	10.5	.931[.896/.966]	.290[.183/.397]	.221[.108/.334]		.794[.743/.846]	.588[.423/.754]	1.31[1.12/1.53]	0.24[0.13/0.45]	
Insomnia		.76 [.69/.82]	diagnostic	24.5	.286[.224/.348]	.913[.846/.980]	.199[.108/.290]		.906[.835/.978]	.303[.240/.365]	3.29[1.49/7.28]	0.78[0.70/0.88]	
			balanced	15.5	.729[.668/.790]	.725[.620/.830]	.454[.332/.576]		.886[.838/.934]	.476[.381/.572]	2.65[1.79/3.92]	0.37[0.29/0.49]	
			screening	9.5	.901[.860/.942]	.333[.222/.444]	.234[.115/.353]		.799[.747/.851]	.533[.384/.682]	1.35[1.14/1.61]	0.30[0.18/0.51]	
Suicidality		.76 [.70/.82]	diagnostic	9.5	.468[.399/.537]	.913[.846/.980]	.381[.285/.477]		.941[.894/.987]	.368[.296/.441]	5.38[2.47/11.72]	0.58[0.50/0.68]	
			balanced	7.5	.645[.579/.711]	.739[.635/.843]	.384[.261/.507]		.879[.827/.931]	.414[.327/.501]	2.47[1.64/3.72]	0.48[0.38/0.61]	
			screening	5	.995[.985/1]	.000[.000/.000]	− .005[-.015/.005]		.745[.694/.797]	.000[.000/.000]	1.00[0.99/1.01]	***	
Appetite Loss		.72 [.66/.79]	diagnostic	7.5	.458[.389/.527]	.913[.846/.980]	.371[.276/.466]		.939[.892/.986]	.364[.292/.436]	5.26[2.42/11.47]	0.59[0.51/0.69]	
			balanced	5.5	.650[.584/.716]	.652[.540/.764]	.302[.172/.432]		.846[.789/.903]	.388[.299/.476]	1.87[1.33/2.62]	0.54[0.42/0.69]	
			screening	2	1[1/1]	.000[.000/.000]	.000[.000/.000]		.746[.695/.798]	***	1.00[1.00/1.00]	***	
Well-Being (recode)		.80 [.74/.85]	diagnostic	32.5	.498[.429/.567]	.913[.846/.90]	.411[.315/.507]		.944[.900/.988]	.382[.308/.456]	5.72[2.63/12.45]	0.55[0.47/0.64]	
			balanced	28.5	.709[.647/.771]	.681[.571/.791]	.390[.264/.516]		.867[.816/.919]	.443[.348/.538]	2.22[1.56/3.17]	0.43[0.33/0.56]	
			screening	22.5	.941[.909/.973]	.406[.290/.522]	.347[.227/.467]		.823[.774/.872]	.701[.559/.842]	1.58[1.30/1.93]	0.15[0.08/0.27]	
Ill Temper		.75 [.68/.81]	diagnostic	17.5	.320[.256/.384]	.928[.867/.989]	.248[.159/.337]		.929[.869/.989]	.317[.253/.381]	4.44[1.86/10.61]	0.73[0.65/0.82]	
			balanced	10.5	.709[.647/.771]	.638[.525/.751]	.347[.218/.476]		.852[.799/.906]	.427[.332/.522]	1.96[1.42/2.71]	0.46[0.35/0.60]	
			screening	6.5	.931[.896/.966]	.232[.132/.332]	.163[.057/.269]		.781[.729/.833]	.533[.355/.712]	1.21[1.06/1.39]	0.30[0.15/0.58]	
Panic		.81 [.75/.87]	diagnostic	26.5	.360[.294/.426]	.913[.846/.980]	.273[.179/.367]		.924[.866/.982]	.327[.260/.393]	4.14[1.89/9.08]	0.70[0.62/0.80]	
			balanced	17.5	.704[.641/.767]	.754[.652/.856]	.458[.339/.577]		.894[.846/.942]	.464[.372/.556]	2.86[1.88/4.37]	0.39[0.31/0.51]	
			screening	12.5	.921[.884/.958]	.507[.389/.625]	.428[.304/.552]		.846[.798/.894]	.686[.558/.813]	1.87[1.47/2.38]	0.16[0.09/0.26]	
Social Anxiety		.71 [.64/.77]	diagnostic	19.5	.340[.275/.405]	.928[.867/.989]	.268[.179/.357]		.933[.876/.990]	.323[.258/.389]	4.72[1.98/11.26]	0.71[0.63/0.80]	
			balanced	12.5	.690[.626/.754]	.623[.509/.737]	.313[.182/.444]		.843[.788/.899]	.406[.312/.499]	1.83[1.33/2.51]	0.50[0.38/0.66]	
			screening	7.5	.906[.866/.946]	.188[.096/.280]	.094[-.007/.195]		.766[.713/.820]	.405[.235/.575]	1.12[0.99/1.26]		0.50[0.26/0.96]
Claustro-phobia		.70 [.63/.77]	diagnostic	13.5	.369[.303/.435]	.913[.846/.980]	.282[.188/.376]		.926[.869/.983]	.330[.263/.396]	4.24[1.93/9.30]		0.69[0.61/0.79]
			balanced	7.5	.645[.579/.711]	.652[.540/.764]	.297[.167/.427]		.845[.788/.902]	.384[.296/.472]	1.85[1.32/2.60]		0.54[0.42/0.70]
			screening	4	.995[.985/1]	.000[.000/.000]	−.005[-.015/.005]		.745[.694/0.797]	.000[.000/.000]	1.00[0.99/1.01]		***
Traumatic Intrusions		.74 [.68/.81]	diagnostic	15.5	.276[.215/.337]	.913[.846/.980]	.189[.098/.280]		.903[.830/.977]	.300[.238/.362]	3.17[1.43/7.03]		0.79[0.71/0.89]
			balanced	9.5	.660[.595/.725]	.725[.620/.830]	.385[.261/.509]		.876[.824/.928]	.420[.332/.509]	2.40[1.62/3.57]		0.47[0.37/0.60]
			screening	5.5	.936[.902/.970]	.319[.209/.429]	.255[.140/.370]		.802[.751/.852]	.629[.469/.789]	1.37[1.17/1.62]		0.20[0.11/0.38]
General Depression	General Health	.74 [.68/.78]	diagnostic	74.5	.405[.318/.492]	.901[.854/.948]	.306[.206/.406]		.765[.661/.869]	.655[.591/.720]	4.09[2.42/6.92]		0.66[0.57/0.77]
			balanced	63.5	.669[.585/.753]	.678[.604/.752]	.347[.235/.459]		.623[.540/.707]	.720[.647/.794]	2.08[1.60/2.70]		0.49[0.37/0.64]
			screening	49.5	.909[.858/.960]	.336[.261/.411]	.245[.154/.336]		.521[.454/.589]	.823[.728/.918]	1.37[1.21/1.55]		0.27[0.15/0.50]
Dysphoria		.71 [.65/.79]	diagnostic	40.5	.298[.217/.379]	.914[.869/.959]	.212[.119/.305]		.734[.610/.857]	.621[.557/.684]	3.47[1.93/6.22]		0.77[0.68/0.87]
			balanced	33.5	.678[.595/.761]	.678[.604/.752]	.356[.244/.468]		.626[.543/.709]	.726[.652/.799]	2.11[1.62/2.73]		0.48[0.36/0.63]
			screening	23.5	.917[.868/.966]	.211[.146/.276]	.128[.047/.209]		.481[.416/.545]	.762[.633/.890]	1.16[1.05/1.28]		0.39[0.20/0.77]
Lassitude		.72 [.66/.78]	diagnostic	21.5	.223[.149/.297]	.928[.887/.969]	.151[.066/.236]		.711[.567/.856]	.600[.537/.663]	3.10[1.60/6.00]		0.84[0.75/0.93]
			balanced	15.5	.694[.612/.776]	.658[.583/.733]	.352[.241/.463]		.618[.536/.699]	.730[.655/.804]	2.029[1.58/2.61]		0.47[0.35/0.62]
			screening	11.5	.942[.900/.984]	.296[.223/.369]	.238[.154/.322]		.516[.450/.582]	.865[.772/.958]	1.34[1.2/1.5]		0.20[0.09/0.42]
Suicidality		.71 [.64/.78]	diagnostic	13.5	.331[.247/.415]	.934[.895/.973]	.265[.172/.358]		.800[.689/.911]	.637[.574/.700]	5.02[2.62/9.60]		0.72[0.63/0.82]
			balanced	8.5	.645[.560/.730]	.697[.624/.770]	.342[.230/.454]		.629[.544/.714]	.712[.639/.784]	2.13[1.62/2.80]		0.51[0.39/0.66]
			screening	5	1[1/1]	.007[-.006/.020]	.007[-.006/.020]		.445[.386/.504]	1[1/1]	1.01[0.99/1.02]		0.000[--/--]
Panic		.72 [.66/.78]	diagnostic	31.5	.140[.078/.202]	.934[.895/.973]	.074[.001/.147]		.628[.446/.810]	.577[.515/.639]	2.12[1.01/4.46]		0.92[0.85/1.00]
			balanced	20.5	.669[.585/.753]	.678[.604/.752]	.347[.235/.459]		.623[.540/.707]	.720[.647/.794]	2.08[1.60/2.70]		0.49[0.37/0.64]
			screening	13.5	.909[.858/.960]	.336[.261/.411]	.245[.154/.336]		.521[.454/.589]	.823[.728/.918]	1.37[1.21/1.55]		0.27[0.15/0.50]
Traumatic Intrusions		.72 [.66/.79]	diagnostic	15.5	.397[.310/.484]	.908[.862/.954]	.305[.206/.404]		.775[.671/.879]	.654[.590/.718]	4.32[2.50/7.45]		0.66[0.57/0.77]
			balanced	11.5	.653[.568/.738]	.711[.639/.783]	.364[.253/.475]		.643[.558/.727]	.720[.648/.792]	2.26[1.71/2.99]		0.49[0.38/0.64]
			screening	5.5	.942[.900/.984]	.191[.129/.253]	.133[.058/.208]		.481[.417/.545]	.805[.676/.935]	1.16[1.07/1.27]		0.30[0.14/0.67]

*Note * J = Youden’s index. *** when specificity is zero, LR- cannot be calculated; when specificity is 1, LR + cannot be calculated; when specificity is zero and sensitivity is 1, NPV cannot be calculated

Regarding the prediction of SF-36 General Health, the highest discrimination value was found for the IDAS-II General Depression (AUC = .74). The balanced cutoff (≥ 64; percentile 53) had comparable specificity (.669 [.59/.75]) and specificity (.678 [.60/.75]). The diagnostic cutoff (≥ 75; percentile 77) corresponded to high specificity (.901 [.85/.95]). The screening cutoff of General Depression for discriminating poor General Health was the same as for discriminating poor Mental Health (≥ 50; percentile 23). While general IDAS-II scales (General Depression and Dysphoria scales) show higher specificity and J index than those computed for MINI diagnoses, all sensitivity values for predicting General Health are higher for MINI diagnoses (see supplementary Table S1).

## Discussion

The present study expands the clinical utility of the Spanish version of the IDAS-II [39] by providing cutoffs to discriminate the diagnoses of the main emotional disorders and to identify poor QoL of those symptoms most associated. Consistent with our hypotheses, the specific IDAS-II scales of Suicidality, Panic, Social Anxiety, Claustrophobia, and Traumatic Intrusions adequately discriminated their corresponding diagnoses (SBD, PD, SAD, Agoraphobia, and PTSD), yet Checking, Ordering, and Cleaning showed poor discriminating values for OCD. Regarding the broad IDAS-II scales, both General Depression and Dysphoria showed adequate discrimination values for MDD. However, in contrast to our second hypothesis, only General Depression scores were adequate to explain GAD. The IDAS-II scales showed higher discrimination for Mental Health-related QoL, than for General Health-related QoL, with General Depression and Dysphoria showing best explanatory ability to determine poor QoL. The results of this study and their application in clinical practice are discussed below.

Regarding the accuracy of the specific IDAS-II scales in discriminating the presence of internalizing disorders, the results were partially consistent with our first hypothesis, as the Trauma Avoidance scale did not show adequate discrimination for predicting PTSD, and neither did Checking, Ordering, and Cleaning for predicting OCD. Several studies suggest additional variables mediating avoidant behaviors and PTSD. For instance, Pineles et al. [64] found that individuals who greatly used avoidant coping strategies were more reactive to trauma reminders and, thus, may be at greater risk of increasing their PTSD symptoms. Serrano-Ibáñez [65] suggest a moderated mediation influence of other additional variables such as guilt and dissociation on the relationship between avoidance and PTSD symptoms. These evidences may suggest an indirect relation of traumatic avoidance of PTSD diagnosis, partially explaining the present results. Interestingly, Traumatic Avoidance and Traumatic Intrusions adequately predicted SBD. This is consistent with previous research showing that the suicide rate is 5.36 times higher in individuals diagnosed with PTSD [66], where intrusion and avoidance symptoms are significantly associated with suicide attempts [67]. This suggests the need to explore SBD symptoms when PTSD is diagnosed, and to treat suicidality when necessary. The poor discrimination found for the Checking, Ordering, and Cleaning scales can be partially explained by the measures used, as the MINI assesses global OCD [48], whereas the IDAS-II assesses specific types of OCD (checking, ordering, and cleaning) [35]. Consequently, MINI diagnoses of OCD do not necessarily involve checking, ordering, or cleaning, implying that some positive OCD cases are false positives for OCD subtype checking, ordering, or cleaning. This could have contributed to the poor discrimination observed for the Checking, Ordering, and Cleaning scales in predicting OCD.

Regarding the broad IDAS-II scales, General Depression showed adequate discrimination for predicting MDD and GAD, and Dysphoria for predicting MDD but not GAD. However, Dysphoria showed adequate ability to predict GAD, as assessed by the MINI in a previous study [46]. The mixed results found for Dysphoria could be partially explained by the use of different samples. While Stasik-O’Brien et al. [46] used a mixed sample (community adults and patients), our sample was exclusively composed of patients. The use of control samples (i.e., community) in clinical studies increases the rate of well-predicted non-diseased individuals and, consequently, the AUC scores [68]. This is consistent with the higher AUC values observed for the IDAS and IDAS-II broad scales in previous studies using mixed samples [45, 46]. Concerning the second hypothesis, General Depression and Dysphoria showed greater AUC values for MDD but not for GAD, as the second-highest AUC values for these scales were found for SBD. This is probably because the Dysphoria scale assesses more depressive symptoms commonly associated with SBD than anxiety symptoms of GAD.

Regarding the accuracy of the IDAS-II in discriminating poor QoL, IDAS-II scores showed a greater ability to explain Mental Health than General Health-related QoL, in line with previous studies [69–71]. Consistent with the third hypothesis, Dysphoria and General Depression showed a better explanatory ability to determine poor Mental Health and General Health-related QoL. The Panic scale showed a particularly high discriminative ability to predict Mental Health-related QoL, with PD being one of the disorders more associated with poor QoL [72–74]. In contrast, other scales (e.g., Euphoria, Mania, Ordering), did not show discriminative ability in predicting QoL. These results are consistent with previous studies that highlighted the importance of evaluating the impact of each symptom separately, as each symptom has a distinct impact on impairment [29, 30].

Concerning clinical utility, the cutoffs provided respond to the demands in clinical practice, allowing adequate discrimination of seven of the eight disorders assessed by IDAS-II scales (MDD, GAD, SBD, PD, SAD, Agoraphobia, and PTSD). Moreover, we provided three cutoffs for screening, research, and diagnostic purposes [46]. Specifically, diagnostic cutoffs can guide the selection of syndrome-specific treatments by accurately identifying internalizing disorders. Unlike traditional diagnostic interviews (e.g., Structured Clinical Interview for DSM [75] and MINI [48]), the IDAS-II evaluates symptom severity in agreement with dimensional approaches. Similarly, QoL cutoffs evaluate QoL as a treatment outcome, in line with recent demands [25, 73]. Assessment of QoL is particularly relevant for internalizing disorders, such as PD, which are significantly associated with poor QoL even after symptom remission [73]. Therefore, treatment should focus on improving the QoL until normal levels are achieved. The IDAS-II may be used to track changes in QoL during treatment, and thus guide evidence-based treatment decisions.

Despite the novel contributions of this study, it has several limitations. First, the use of a non-probability sampling procedure may limit the generalizability of the results, as the cutoffs may not be equally accurate in classifying individuals from other samples with different idiosyncratic characteristics [76]. However, assuming slight differences, we believe that our sample can be representative of those patients targeted by the use of these cutoffs, as it includes patients from public and private centers at different stages of treatment and with different levels of symptom severity. Second, the larger proportion of women in our sample limits the generalizability of our findings to a broader population. Nevertheless, the proportion of women in our sample was similar to that reported in a previous study that developed diagnostic cutoffs for the first version of IDAS [46]. The higher proportion of women is also consistent with the higher prevalence of depression, anxiety, and bipolar disorder in women [1]. Nevertheless, future studies should replicate analyses of samples with similar proportions of men and women. Third, the instrument used to evaluate clinical diagnoses does not assess specific types of OCD covered by the IDAS-II (checking, ordering, and cleaning), which probably interferes with the discrimination values found for the OCD scales of the IDAS-II. Future studies should incorporate measures to specifically assess checking, ordering, and cleaning OCD. Fourth, as our sample included very few individuals diagnosed with BID and BIID, it would be convenient to increase the number of participants with these disorders in future studies to develop diagnostic and QoL cutoffs for bipolar disorders. Similarly, future studies with broader samples, including participants with and without comorbidity, would allow for the analysis of whether the presence of comorbidity influences the discriminative ability of the instrument to detect specific disorders. This approach would provide a deeper understanding of the potential impact of comorbid conditions on the diagnostic accuracy and utility of the instrument.

## Conclusions

The present study provides diagnostic and QoL cutoffs for the IDAS-II, thereby expanding the clinical utility of this measure in clinical practice and research. By providing diagnostic cutoffs, the instrument gathers both novel dimensional approaches and traditional categorical approaches that rely on patients’ diagnoses to determine the choice of treatment. Similarly, the IDAS-II can also be employed to meet more recent clinical practices advocating QoL assessment and intervention in the treatment of mental health problems owing to QoL cutoffs. In summary, the present study increased the applicability of the IDAS-II, making it a comprehensive, detailed, and versatile self-report to assess internalizing symptoms.

### Electronic supplementary material

Below is the link to the electronic supplementary material.

Supplementary Material 1

## Data Availability

The dataset analyzed during the current study is available in the Open Science Framework repository: https://osf.io/2ez5h/?view_only=c370c1d7f50e457794bc5230fd47ddd8.

## Abbreviations

BID: Bipolar I Disorder
BIID: Bipolar II Disorder
GAD: Generalized Anxiety Disorder
HiTOP: Hierarchical Taxonomy of Psychopathology
IDAS: Inventory of Depression and Anxiety Symptoms
MDD: Major Depressive Disorder
MINI: Mini-International Neuropsychiatric Interview
NPV: Negative Predictive Value
OCD: Obsessive-Compulsive Disorder
PD: Panic Disorder
PPV: Positive Predictive Value
PTSD: Post-Traumatic Stress Disorder
QoL: Quality of Life
ROC: Receiver Operating Characteristic
SAD: Social Anxiety Disorder
SF-36: Short Form-36 Health Survey
