# Supplementary material for: Discriminative capacity of the Spanish version of the Inventory of Depression and Anxiety Symptoms-II (IDAS-II) for detecting DMS-5 specific disorders and poor quality of life in a clinical sample
Source: Health Qual Life Outcomes. 2024 Jul 18;22:56. doi: 10.1186/s12955-024-02270-x (PMC11256423; doi:10.1186/s12955-024-02270-x)
Supplement: Supplementary file 1 — Supplementary Material 1 [file 12955_2024_2270_MOESM1_ESM.docx]

**Table S1.**

*Sensitivity, Specificity, J index, PPV, NPV, and LR values of MINI diagnoses predicting SF-36 Mental Health and General Health.*

| MINI diagnosis | Predicting T<35 | Sensitivity | Specificity | J | PPV | NPV | LR+ | LR- |
| --- | --- | --- | --- | --- | --- | --- | --- | --- |
| GAD | Mental Health | .606 [.539/.673] | .797 [.702/.892] | .403 [.287/.519] | .898 [.847/.949] | .407 [.325/.490] | 2.985 [1.846/4.827] | .494 [.401/.609] |
|  | General Health | .669 [.585/.753] | .631 [.554/.708] | .300 [.186/.414] | .591 [.508/.673] | .705 [.629/.782] | 1.813 [1.422/2.311] | .525 [.396/.695] |
| MDD | Mental Health | .586 [.518/.654] | .884 [.808/.960] | .470 [.369/.571] | .937 [.895/.979] | .421 [.340/.501] | 5.052 [2.607/9.789] | .468 [.389/.563] |
|  | General Health | .603 [.516/.690] | .645 [.569/.721] | .248 [.132/.364] | .575 [.489/.661] | .671 [.595/.747] | 1.699 [1.312/2.200] | .616 [.480/.790] |
| SAD | Mental Health | .182 [.129/.235] | .956 [.908/1.004] | .138 [.066/.210] | .924 [.842/1.006] | .284 [.226/.342] | 4.136 [1.326/12.906] | .856 [.788/.929] |
|  | General Health | .215 [.142/.288] | .907 [.861/.953] | .122 [.035/.209] | .648 [.500/.796] | .592 [.529/.655] | 2.312 [1.266/4.221] | .865 [.778/.963] |
| SBD | Mental Health | .050 [.020/.080] | .986 [.958/1.014] | .036 [-.005/.077] | .913 [.748/1.079] | .261 [.208/.314] | 3.571 [0.451/28.274] | .963 [.924/1.005] |
|  | General Health | .067 [.022/.112] | .980 [.958/1.002] | .047 [-.003/.097] | .727 [.466/.989] | .569 [.509/.629] | 3.350 [0.916/12.247] | .952 [.903/1.004] |
| PD | Mental Health | .227 [.169/.285] | .899 [.828/.970] | .126 [.034/.218] | .869 [.778/.960] | .283 [.224/.343] | 2.248 [1.063/4.750] | .860 [.771/.959] |
|  | General Health | .306 [.224/.388] | .895 [.846/.944] | .201 [.106/.296] | .699 [.575/.822] | .618 [.554/.683] | 2.914 [1.705/4.982] | .775 [.681/.883] |
| Agorafobia | Mental Health | .227 [.169/.285] | .971 [.931/1.011] | .198 [.128/.268] | .958 [.902/1.015] | .299 [.239/.359] | 7.828 [1.952/31.387] | .796 [.731/.867] |
|  | General Health | .298 [.217/.379] | .921 [.878/.964] | .219 [.127/.311] | .750 [.628/.873] | .622 [.559/.686] | 3.772 [2.054/6.927] | .762 [.673/.864] |
| OCD | Mental Health | .369 [.303/.435] | .826 [.737/.915] | .195 [.084/.306] | .862 [.789/.934] | .308 [.241/.374] | 2.121 [1.230/3.656] | .764 [.657/.888] |
|  | General Health | .339 [.255/.423] | .724 [.653/.795] | .063 [-.047/.173] | .494 [.387/.602] | .579 [.509/.649] | 1.228 [0.859/1.757] | .913 [.777/1.072] |
| PTSD | Mental Health | .251 [.191/.311] | .913 [.846/.980] | .164 [.075/.253] | .895 [.815/.974] | .293 [.232/.354] | 2.885 [1.296/6.424] | .820 [.736/.914] |
|  | General Health | .306 [.224/.388] | .868 [.814/.922] | .174 [.076/.272] | .649 [.525/.772] | .611 [.546/.676] | 2.318 [1.423/3.777] | .800 [.700/.914] |

*Note*: J = Youden’s index; PPV = predictive positive value; NPV = negative predictive value; LR+ = positive likelihood ratio; LR- = negative likelihood ratio.
